# Antitumor, Antioxidant and Antimicrobial Studies of Substituted Pyridylguanidines

**DOI:** 10.3390/molecules180910378

**Published:** 2013-08-27

**Authors:** Muhammad Said, Amin Badshah, Naseer Ali Shah, Hizbullah Khan, Ghulam Murtaza, Boris Vabre, Davit Zargarian, Muhammad Rashid Khan

**Affiliations:** 1 Department of Chemistry, Quaid-i-Azam University, Islamabad 45320, Pakistan; 2 Department of Chemistry, Abdul Wali Khan University, Mardan 23200, Pakistan; 3 Department of Biochemistry, Quaid-i-Azam University, Islamabad 45320, Pakistan; 4 Department of Chemistry, University of Science and Technology, Bannu 28100, Pakistan; 5 Départment de Chimie, Université de Montréal, Montréal, QC H3C3J7, Canada

**Keywords:** guanidine, antitumor, antioxidant, antimicrobial assay

## Abstract

A series of *N*-pivaloyl-*N′*-(alkyl/aryl)-*N″*-pyridylguanidine of general formula C_4_H_9_CONHC(NR^1^R^2^)NPy have been synthesized and characterized using elemental analysis, FT-IR, multinuclear NMR spectroscopy, and in the case of compounds **7** and **11**, by single crystal X-ray diffraction (XRD). The synthesized guanidines were tested for antitumor activities against potato tumor, and showed excellent inhibition against *Agrobacterium tumefaciens* (AT10)-induced tumor. The antioxidant and antimicrobial activities of these new compounds against various bacterial and fungal strains were also investigated.

## 1. Introduction

Treatment of cancer and infectious diseases faces serious difficulties due to the development of resistance to current anticancer/antibiotic drugs. Therefore the discovery and development of new anticancer/antibiotic agents is a high priority in biomedical research. There are a large number of anticancer/antimicrobial agents of natural and synthetic origin having guanidine functionalities. Guanidines are physiologically active substances possessing a wide spectrum of activities including anticancer [1,2], antidiabetic [3,4], antiviral, anti-inflammatory [5], antibiotic [6,7], antileishmenial [8,9,10,11,12,13,14,15,16], antiprotozoal, antihistaminic and antihypertensive [17] properties. Such a diverse range of biochemical behavior can be attributed to their flexible structure and the three nitrogen atoms of a guanidine moiety which makes it possible to bind to various substituents. In case of drugs having low penetration through different membranes in body, the introduction of a guanidinium group into their molecular architecture increases the ability of these molecules to cross biological barriers and thus enhances their biological activities [18,19].

In the human body cancer is a multi-step process that often involves the inactivation of tumor suppressor genes or activation of oncogenes [20], caused by many factors. Other than genetic mutation, different chemical species which interfere with the enzyme’s structure or activity are also responsible for cancer. Reactive oxygen species (ROS), produced as by-products of metabolic reactions in living organisms which initiate toxic oxidative reactions in biomolecules, causes oxidative stress. A state of oxidative stress has deleterious effects on almost all tissues and can initiate or enhance the rate of pathological conditions such as neurodegeneration, inflammation, aging process, cancer and cardiovascular diseases [21,22,23]. Heterocyclic guanidine derivatives have shown remarkable antioxidant properties for reducing the oxidative stress, induced in blood serum and brain tissue by superoxide dismutase and catalase enzymes during ischemia-reperfusion, causing restoration of blood supply and having neuroprotective role [24]. Naturally occurring polycyclic guanidine extracted from the sponge *Monanchora unguifera* [15] showed significant antitumor properties against several human cancer cell lines. A variety of guanidines have been synthesized and screened for their antitumor behavior [25,26] showing remarkable results. They are potent anticancer agents against breast, lung, colon, oral, cervix, prostate and ovarian cancer in humans [27,28]. Guanidines having a pyrrolidine moiety as well as 2-aminoimidazole rings have been studied for cytotoxic activity against several human tumor cell lines, exhibiting good inhibitory activities [29]. Guanidine derivatives of thiophene-fused tetracyclic analogues of ametantrone have good cytotoxic activity against variety of tumor cell lines including isogenic drug-resistant counterparts [30].

Guanidines have also shown promising antibacterial and antifungal properties [31,32,33,34]. Synthetically substituted guanidines are mostly prepared by two strategies *i.e.*, guanylation and guanidinylation. In guanylation, a new guanidine moiety is generated in a molecule while in guanidinylation a new substituent is placed on an already present guanidine moiety [35,36,37]. Keeping in view the potential antitumor/antioxidant activity of guanidines [38], herein we report the synthesis of a series of pyridylguanidines derivatives. The antibacterial and antifungal activities of the synthesized compounds are also investigated.

## 2. Results and Discussions

### 2.1. Synthesis and Characterization

**Scheme 1:**
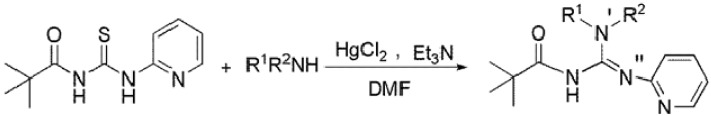
Synthesis of substituted pyridylguanidines **1**–**12**.

The guanylation approach used for synthesis of the new guanidines is illustrated in Scheme 1. Thiourea reacts with primary and secondary amines in the presence of triethylamine and mercury(II) chloride and produces guanidines. The HgCl_2_ plays the role of a Lewis acid to further activate the electrophilic carbon of thiourea which then undergoes a nucleophilic attack by the amine. The formation of the new C-N bond and the expulsion of black HgS byproduct lead to the formation of the new guanidine. This reaction is carried out at low temperature (0 °C) because it is highly exothermic. All the new compounds were colorless crystalline solids and synthesized in good yields; they are all soluble in common organic solvents. The identities of the products were established by elemental analysis, IR spectroscopy, multinuclear NMR (^1^H and ^13^C) spectroscopy and single crystal X-ray diffraction studies of selected examples. The agreement between experimental and calculated values for elemental analysis confirmed the successful synthesis and purity of desired compounds. 

R^1^ = phenyl (**1**), 2-chlorophenyl (**2**), 3-chlorophenyl (**3**), 4-chlorophenyl (**4**), 2-methoxyphenyl (**5**), 4-tolyl (**6**), 2-fluorophenyl (**7**), *n*-propyl (**8**, **12**), *iso*-propyl (**9**), *iso*-butyl (**10**) and 2-pyridyl (**11**), R^2^ = H (**1–11**), *n*-propyl (**12**).

#### 2.1.1. IR Spectra

In the IR spectra, the C=O peaks appear in the range of 1,617–1,658 cm^−1^. These appear at relatively lower frequencies than in normal amides due to intramolecular H-bonding between N′-H and O which weakens the C=O bond. There are two NH peaks in IR spectra of compounds **1**–**11**, one for N-H in range of 3,417–3,437 cm^−1^ and other for N′-H in range of 3,239–3,269 cm^−1^ confirming intramolecular H-bonding, while **12** has only one NH peak at 3,439 cm^−1^.

**Table 1:** Characteristic pyridylguanidine resonances by NMR spectroscopy.

Compound	R^1^	R^2^	^1^H			^13^C	
			NH	NH	CN_3_		C=O
**1**	Phenyl	H	11.39	14.52	161.6		180.6
**2**	2-chlorophenyl	H	11.91	14.49	161.2		180.2
**3**	3-chlorophenyl	H	11.32	14.46	161.3		180.9
**4**	4-chlorophenyl	H	11.26	14.48	161.3		180.7
**5**	2-methoxyphenyl	H	11.93	14.54	161.8		179.9
**6**	4-tolyl	H	11.36	14.53	161.8		180.6
**7**	2-fluorophenyl	H	11.79	14.47	161.3		180.5
**8**	*n*-propyl	H	9.02	14.59	162.7		180.3
**9**	*iso*-propyl	H	8.99	14.57	162.7		180.4
**10**	*iso*-butyl	H	8.98	14.56	162.7		180.4
**11**	2-pyridyl	H	12.08	14.36	161.3		180.0
**12**	*n*-propyl	*n*-propyl	12.27	----	161.8		176.7

#### 2.1.2. NMR Spectra

The ^1^H-NMR spectra are normal, showing two broad NH peaks as singlets in the case of trisubstituted guanidines **1**–**11** and one broad peak as singlet in case of the tetrasubstituted guanidine **12**. Aromatic protons are observed as multiplets while the (CH_3_)_3_ group appears as a singlet in the 1.12–1.22 ppm range. In the ^13^C spectra the carbonyl carbons are observed at ca. 177–181 ppm, while the (CN_3_) carbons of the guanidine moiety give signals at ca. 161–163 ppm (Table 1). The aromatic carbons are observed at normal chemical shift while the tertiary carbons of the pivaloyl group give signals at 40.5 ± 0.2 ppm and primary carbons are observed at 27.2 ± 0.1 ppm in all compounds.

#### 2.1.3. X-ray Diffraction Analysis

Rod-shaped colourless crystals of **7** and **11** were grown by slow evaporation of their methanol solutions. Crystal data and structure refinement parameters are given in Table 2, while selected bond lengths (Å), bond angles (°) and torsion angles (°) are given in Table 3.

**Table 2:** Crystal data and structure refinement parameters for compounds (**7** and **11**).

Crystal parameters		7	11
Empirical formula		C_17_H_19_N_4_OF	C_16_H_19_N_5_O
Formula weight		314.36	297.35
Temperature (K)		296	200
Wavelength (Å)		1.54178	1.54178
Crystal system		Monoclinic	Monoclinic
Space group		P2(1)/n	P2(1)/n
Unit cell dimensions	a (Å)	10.7110(2)	6.00430(10)
	b(Å)	9.9954(2)	16.9550(2)
	c(Å)	15.2679(4)	15.2900(2)
	α(°)	90	90
	β(°)	91.0430(10)	92.4800(10)
	γ(°)	90	90
V (Å3), Z		1634.32(6),4	1555.11(4),4
Density (calcd) (g/cm^3^)		1.278	1.270
Crystal size(mm^3^)		0.10 × 0.08 × 0.08	0.14 × 0.12 × 0.10
Index ranges		−13 <= h <= 12 −12 <= k <= 12 −18 <= l <= 17	−7 <= h <= 7 −20 <= k <= 20 −18 <= l <= 18
F(000)		664	632
Total reflections		21339	20265
Independent reflections		3214 [Rint= 0.045]	3032[Rint= 0.036]
R indices (all data)		R1 = 0.0516 wR2 = 0.1220	R_1_ = 0.0464, wR_2_ = 0.1154
Final R indices [I > 2σ(I)]		R1 = 0.0445, wR2 = 0.1141	R_1_ = 0.0402, wR_2_ = 0.1093
Goodness-of-fit		1.048	1.042
Theta range for data collection (°)		5.00 to 72.64	3.89 to 72.53

**Table 3:** Selected bond lengths, bond angles and torsion angles for compounds **7** and **11**.

Compound	Bond lengths (Å)		Bond angles (°)	Torsion angles (°)
**7**	C5-O1 1.2306(17)	C6-N1 1.4034(18)	N1-C6-N2 114.25(12)	O1-C5-N1-C6 1.0(2)
	C6-N2 1.3613(17)	C6-N3 1.2929(18)	N1-C6-N3 123.83(12)	C5-N1-C6-N2 4.8(2)
	C5-N1 1.3644(18)	C7-N3 1.3985(17)	N2-C6-N3 121.92(13)	C5-N1-C6-N3 -175.45(14)
	C12-N2 1.4027(17)	C2-C5 1.526(2)	C5-N1-C6 128.79(12)	C6-N3-C7-N4 -5.5(2)
**11**	C5-O1 1.2263(15)	C5-N1 1.3653(15)	N1-C6-N2 123.91(11)	O1-C5-N1-C6 0.2(2)
	C6-N1 1.4017(15)	C6-N2 1.2918(15)	N1-C6-N3 114.67(10)	C5-N1-C6-N2 175.27(12)
	C6-N3 1.3623(15)	C7-N3 1.4003(15)	N2-C6-N3 121.42(11)	C5-N1-C6-N3 -5.09(18)
	C12-N2 1.3925(15)	C2-C5 1.5309(16)	C5-N1-C6 129.02(10)	C6-N3-C7-N4 177.02(12)

**Figure 1:**
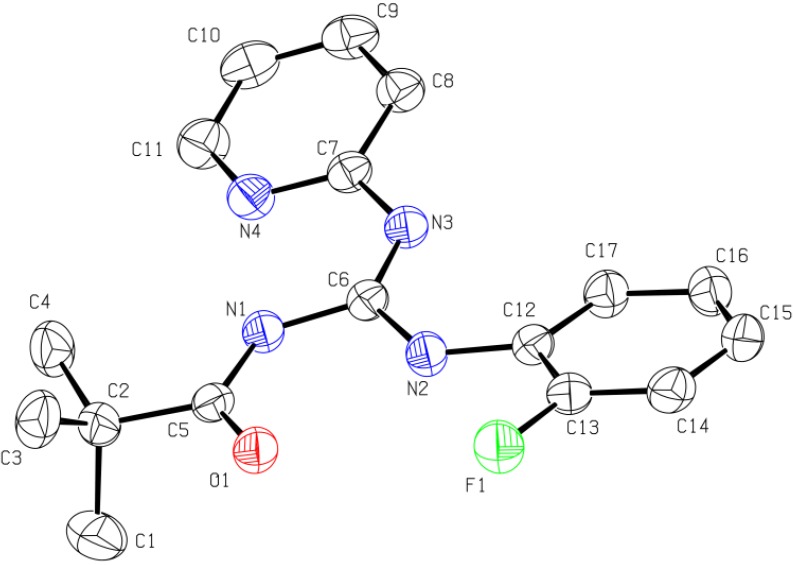
ORTEP diagram for *N*-pivaloyl-*N′*-(2.fluorophenyl)-*N″*-pyridylguanidine (**7**). Thermal ellipsoids are shown at the 50% probability level. Hydrogen atoms are omitted for clarity.

**Figure 2:**
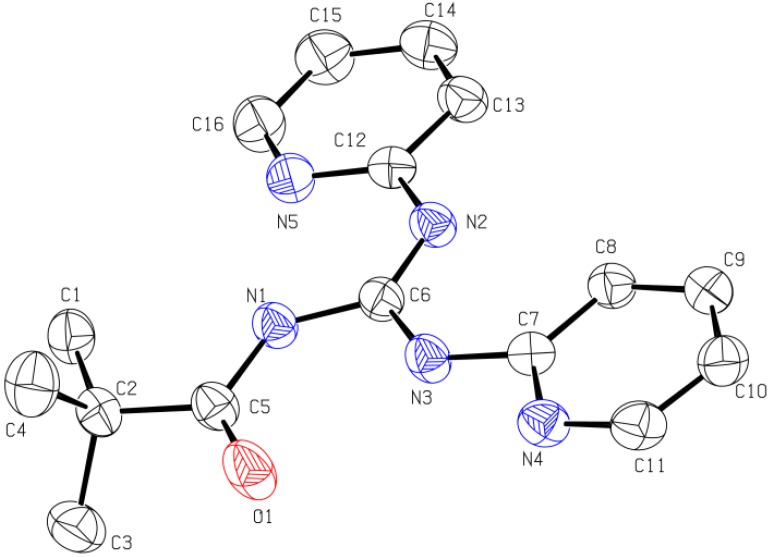
ORTEP diagram of *N*-pivaloyl-*N′*,*N″*-dipyridylguanidine (**11**). Thermal ellipsoids are shown at the 50% probability level. Hydrogen atoms are omitted for clarity.

The single crystal X-ray results show that both compounds crystallize in the monclinic P2(1)/n space group with Z = 4. The guanidine core (NHC(=N)NH) in the molecule is perfectly planar and the sum of angles around the central carbon atom is 360°. The planarity of the guanidine moiety is due to the strong electron delocalization in the CN_3_ unit. The C-N bond lengths in CN_3_ unit are in between those of a double bond (C=N, 1.25–1.28 Å) [39] and a single bond (C-N, 1.45–1.47 Å) [40]. Furthermore, the torsion angles in both compounds indicate that the carbonyl group, guanidine moiety, pyridyl ring, 2-fluorophenyl group (in **7**) and second pyridyl ring (in **11**) are coplanar, creating strong resonance in each molecule. The X-ray results also indicate two intramolecular hydrogen bonds in each molecule. One such interaction is that between N′-H and oxygen atom of carbonyl group forming a six-membered ring, which is commonly observed in this class of compounds [41], while the other involves N-H and N of the pyridyl group. These intramolecular hydrogen bonds are responsible for keeping the carbonyl group, the guanidine unit and the two aryl rings in a plane in the solid state. The single crystal XRD diagrams of **7** and **11** are given in Figure 1 and Figure 2 respectively. 

The two pyridyl rings (in **11**) are crystallographicaly different from each other because one ring is closer in space to carbonyl group than the other. Full details of the XRD results are available free of charge from the The Cambridge Crystallographic Data Centre via www.ccdc.cam.ac.uk/data_request/cif under deposition numbers CCDC 929909 for **7** and CCDC 929908 for **11**.

### 2.2. Biological Studies

#### 2.2.1. Potato Disc Anti-Tumor Assay

A potato disc anti-tumor assay (grown gall tumor inhibition assay) was conducted to test the anticancer behavior of the synthesized compounds. *Agrobacterium tumefaciens* (strain AT10) by its tumor inducing plasmids induces the plant tumor known as grown gall tumor. The protocol reported by Turker and Camper [42] was followed for the potato disc anti-tumor assay. The percentages of tumor inhibition caused by the synthesized compounds are given in Table 4.

**Table 4:** Antitumor activity of synthesized guanidines.

Compound	Average number of tumors per disc	% Inhibition of tumors
**1**	2.0	73
**2**	1.5	80
**3**	2.0	73
**4**	3.0	60
**5**	2.0	73
**6**	1.5	80
**7**	2.0	73
**8**	3.0	60
**9**	2.0	73
**10**	2.0	73
**11**	3.0	60
**12**	1.5	73
**AT10**	7.5	---
**Blank**	00	100

^a^ Potato disc antitumor assay, concentration: 500 µg/mL in DMSO; ^b^ More than 20% tumor inhibition is significant; ^c^ Data represents mean value of 12 replicates.

The results indicated that all of the compounds have significant anti-tumor activity. Compounds **2** and **6** have shown the highest activity, 80% compared to the standard vincristine taken as 100%. It is observed that the aryl substituted pyridylguanidines are more potent anti-tumor agents compared with alkyl substituted compounds but the presence of an alkyl group on the phenyl ring enhances the activity. The activity increases among the aryl substituted pyridylguanidines with changes in the position of the substituent on the phenyl ring from the *para* to the *ortho* position.

**Figure 3:**
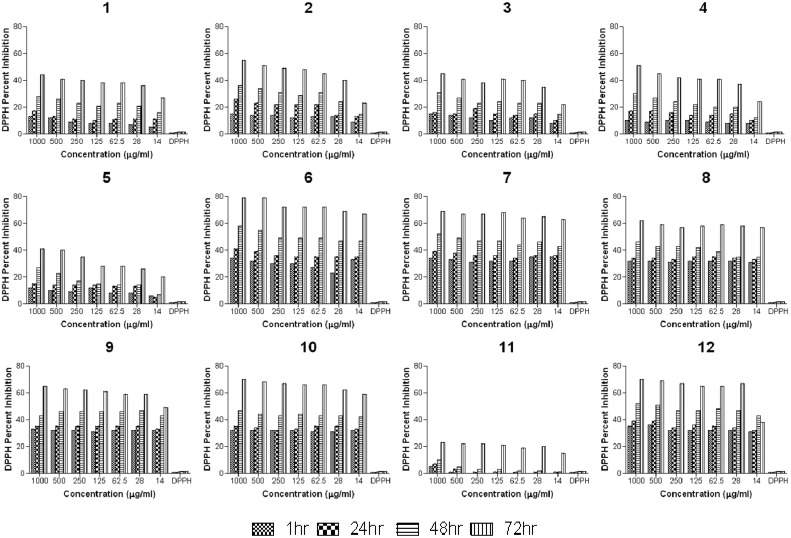
Percent scavenging of DPPH by compounds **1**–**12** at different concentrations (1000, 500, 250, 125, 62.5, 28 and 14 µg/mL) after 1, 24, 48 and 72 h. DPPH without sample was used as control.

#### 2.2.2. Anti-Oxidant Study

The anti-oxidant behavior of the synthesized compounds was investigated by the reported method of Siraj *et al*. [43] with slight modifications. The percent scavenging of DPPH is shown in Figure 3. The results indicate that the scavenging of DPPH by the tested compounds is time dependent and a relatively slow process. It is also observed that scavenging of DPPH by pyridylguanidines can be correlated to the substituent attached at N′ position. Generally the presence of electron donor substituent such as alkyl group enhances the antioxidant property while electron withdrawing aryl group suppresses the DPPH scavenging ability. Among the aryl substituted pyridylguanidines, the scavenging ability is remarkably improved in the presence of the electron donor group on the phenyl ring.

#### 2.2.3. Antifungal Activity

The synthesized guanidines were investigated for their antifungal activity against five fungal strains: *A. flavus*, *A. niger*, *F. solanai*, *M. specie* and *A. fumagatus*. Susceptibility test was performed by using Agar tube dilution method [44]. The results are summarized in Table 5. The overall results indicated that all compounds have insignificant antifungal activities but the aryl substituted pyridylguanidines specially having substituents at ortho and para position on the phenyl ring such as **2**, **4**, **5**, **6** and **7** have good to significant activity against *A. niger* and *F. solanai*.

**Table 5:** *In vitro* antifungal activity of synthesized guanidines and standard drug.

Compound	Mean Values of Percent Growth Inhibition				
	*A. flavus*	*A. niger*	*F. solani*	*M. species*	*A. fumagatus*
**1**	10 ± 1.5	12.5 ± 2.0	20 ± 1.0	---	47.5 ± 1.5
**2**	---	72.5 ± 1.5	62.5 ± 2.5	---	5 ± 0
**3**	---	2.5 ± 1.5	20 ± 2.0	---	---
**4**	---	79 ± 1.0	32.5 ± 1.5	25 ± 1.0	---
**5**	---	66.5 ± 1.5	65.3 ± 1.7	---	42.5 ± 2.4
**6**	---	72.5 ± 0.5	62.5 ± 0.5	5 ± 0.5	55 ± 1.0
**7**	---	70 ± 2.0	---	50 ± 1.0	5 ± 1.0
**8**	64.5 ± 1.5	42.5 ± 2.5	62.5 ± 1.5	40 ± 2	20 ± 0.5
**9**	---	32.5 ± 0.5	40 ± 2	12.5 ± 0.5	12.5 ± 0.5
**10**	---	27.5 ± 0.5	47.5 ± 1.5	---	20 ± 1
**11**	---	---	25 ± 1.0	---	30 ± 2
**12**	---	---	39.5 ± 1.5	---	10 ± 0
**Terbinafine**	100	100	100	100	100
**Vehicle control**	---	---	---	---	---

(a) --- Shows no activity; (b) *In vitro* agar tube dilution method; (c) concentration: 12 mg/mL of DMSO.

#### 2.2.4. Antibacterial Activity

The synthesized compounds were tested against six bacterial strains; two Gram-Positive (*M. luteus* and *S. aureus*) and four Gram-negative (*E. coli*, *E. aerogenes*, *B. bronchiseptica* and *K. pneumonia*). The agar well-diffusion method was used for the determination of antibacterial activity. The mean zone of inhibition less than 9 mm is considered as no activity, represented as “----”. The antibacterial activity is measured on the basis of zone of inhibition and minimum inhibition concentration compared with standard drug Cefixime and Roxythromycine. The mean zone of inhibition is given in Table 6 while the MIC is given in Table 7. The results indicate that the overall antibacterial activity of synthesized guanidines is insignificant. Compounds **6** and **10** have moderate activity against *E. coli*.

**Table 6:** *In vitro* antibacterial activity (mean zone of inhibition) of synthesized guanidines and standard drugs.

Compound	Mean zone of inhibition (mm)					
	*M. luteus*	*S. aureus*	*K. pneumoniae*	*E. aerogenes*	*E. coli*	*B. bordetella*
**1**	---	---	---	---	---	---
**2**	15 ± 0.5	12 ± 0.8	---	---	15 ± 1.0	16 ± 0.6
**3**	---	---	---	---	---	---
**4**	---	---	---	---	---	---
**5**	---	---	---	---	---	---
**6**	---	---	---	---	18 ± 1.2	---
**7**	---	---	---	---	---	---
**8**	15 ± 1.5	15 ± 0.5	---	---	---	13 ± 1.5
**9**	14 ± 0.6	13 ± 1.2	---	12 ± 0.2	12 ± 0.4	---
**10**	15 ± 0.5	15 ± 0.7	13 ± 0.4	14 ± 0.8	17 ± 1.5	15 ± 0.7
**11**	---	---	---	---	---	---
**12**	12 ± 0.8	12 ± 0.6	12 ± 0.4	---	16 ± 0.8	---
**Cefixime**	25 ± 1.0	22 ± 0.6	22 ± 0.5	23 ± 1.0	25 ± 1.0	20 ± 0.5
**Roxythromycine**	30 ± 1.5	25 ± 1	30 ± 2.0	25 ± 1.0	26 ± 2.0	25 ± 1.0

**Table 7:** Effective Minimum Inhibition Concentration (MIC) of synthesized guanidines against different bacterial strains.

Compound	Minimum inhibitory concentration (MIC) mg/mL DMSO					
	*M. luteus*	*S. aureus*	*K. pneumoniae*	*E. aerogenes*	*E. coli*	*B. bordetella*
**1**	---	---	---	---	---	---
**2**	0.0312	0.0078	---	---	0.0019	0.25
**3**	---	---	---	---	---	---
**4**	---	---	---	---	---	---
**5**	---	---	---	---	---	---
**6**	---	---	---	---	0.25	---
**7**	---	---	---	---	---	---
**8**	1	1	---	---	---	1
**9**	0.25	0.0156	---	0.5	1	---
**10**	0.5	0.25	1	1	0.5	0.5
**11**	---	---	---	---	---	---
**12**	0.5	0.125	0.0019	---	0.0312	---

(a) --- MIC not determined which is more than 1mg/mL; (b) agar well-diffusion method, 24 h incubation.

## 3. Experimental

### 3.1. General

All reactions were carried out under ambient atmosphere. The following reagents were purchased from commercial sources and used as received: aniline, 2-chloroaniline, 3-chloroaniline, 4-chloroaniline, *o*-anisidine, *p*-toluidine, 2-fluoroaniline, *n*-propylamine, *iso*-propylamine, *iso*-butylamine and 2-aminopyridine were obtained from Fluka (Karachi, Pakistan); Mercury(II) chloride and potassium thiocyanate were obtained from Aldrich (Lahore, Pakistan). Various solvents such as acetone, ethanol, methanol, chloroform, dichloromethane, ethyl acetate, dimethylformamide and *n*-hexane were of analytical grade and were obtained from Merck (Karachi, Pakistan) or Fluka. These solvents were dried and purified by standard methods [45]. Melting points were determined in a capillary tube using elector-thermal melting point apparatus model MP-D Mitamura Riken Kogyo (Kyoto, Japan). Elemental analysis were performed on Fisons EA1108 CHNS analyzer and used as a tool for the purity check. Infrared spectra were recorded as KBr discs on a Bio-Rad Elmer 16 FPC FT-IR. ^1^H- (300 MHz) and ^13^C-NMR (75 MHz) were recorded at 25 °C on a Bruker AV300 NMR instrument using C_6_D_6_ as solvent. ^1^H and ^13^C chemical shifts are reported in ppm downfield of TMS and referenced against the residual C_6_D_6_ signals (7.16 ppm for ^1^H and 128.06 for ^13^C) [46]. The splitting of proton resonances in the NMR spectra are indicated as s = singlet, d = doublet, t = triplet, dd = doublet of doublet, sext = sextet and m = multiplet (showing a complex pattern). The crystallographic data for the compounds was collected on Bruker Microstar generator equipped with a Kappa Nonius goniometer and platinum 135 detector. Cell refinement and data reduction were done using SAINT [47]. The space group was confirmed by XPREP routine [48] in the program SHELXTL [49]. The structure was solved by direct method and refined by full-matrix least-squares on F^2^ with SHELX-97 [50,51].

### 3.2. Synthesis

*N-Pivaloyl-N′-phenyl-N′′-pyridylguanidine* (**1**). To a solution of *N*-pivaloyl-*N′*-pyridylthiourea (2.37 g, 10 mmol) in DMF (20 mL) at room temperature were added triethylamine (2.8 mL, 20 mmol) and aniline (1.0 mL, 10 mmol). The mixture was cooled to 273 K and stirred for 5 min, then mercury(II) chloride (2.72 g, 10 mmol) was added and the mixture stirred vigorously. After a few minutes the suspension became black as a result of the formation of HgS. Stirring was continued at room temperature for 12 h and the progress of reaction was monitored by TLC. After completion of the reaction, dichloromethane (20 mL) was added and the suspension was filtered through a sintered glass funnel to remove the HgS formed during the reaction. The solvent was evaporated from the filtrate under reduced pressure. The residue was dissolved in CH_2_Cl_2_ (20 mL) and the solution was extracted with water (4 × 30 mL). The CH_2_Cl_2_ fraction was collected and dried over anhydrous MgSO_4_. The CH_2_Cl_2_ was evaporated and the crude product was purified by column chromatography on silica gel using *n*-hexane/ethyl acetate 10:1 [52]. Yield: 2.07 g, 70%. Colourless crystals. M.p. 62–63 °C. FT-IR (KBr, cm^−1^): 3413, 3245, 3128, 3043, 2988, 1634, 1528, 1453, 1378, 1203, 928, 749. ^1^H-NMR: δ 1.14 (s, 9H, COC(CH_3_)_3_), 6.38–6.43 (m, 1H, Ar-H), 6.90–6.96 (m, 1H, Ar-H), 7.03–7.12 (m, 2H, Ar-H), 7.18–7.23 (m, 2H, Ar-H), 7.86–7.88 (m, 1H, Ar-H), 7.97–8.00 (m, 2H, Ar-H), 11.39 (s, 1H, NH), 14.52 (s, 1H, NH). ^13^C-NMR: δ 27.2 (3C, COC(CH_3_)_3_), 40.6 (COC(CH_3_)_3_), 117.0, 121.5 (2C), 122.4, 123.5, 129.0 (2C), 138.2, 139.4, 145.2, 147.5 (aromatic-C), 161.6 (CN_3_), 180.6 (C=O). Anal. Calcd. for C_17_H_20_N_4_O (296.37): C, 68.89; H, 6.80; N, 18.90. Found: C, 68.71; H, 6.84; N, 18.79%. 

*N-Pivaloyl-N′-(2-chlorophenyl)-N′′-pyridylguanidine* (**2**). Compound **2** was prepared and purified in the same way as **1**, using *N*-pivaloyl-*N′*-pyridylthiourea (2.37 g, 10 mmol), 2-chloroaniline (1.1 mL, 10 mmol), triethylamine (2.8 mL, 20 mmol) and mercury(II) chloride (2.72 g, 10 mmol). Yield: 2.41 g, 73%. Colourless crystals. M. p. 78–79 °C. FT-IR (cm^−1^): 3418, 3269, 3157, 3058, 2984, 1638, 1537, 1458, 1386, 1239, 935, 763. ^1^H-NMR: δ 1.14 (s, 9H, COC(CH_3_)_3_), 6.40–6.43 (m, 1H, Ar-H), 6.64–6.68 (m, 1H, Ar-H), 7.00–7.02 (m, 1H, Ar-H), 7.07–7.13(m, 2H, Ar-H), 7.20 (dd, 1H, *^3^J* = 7.9 Hz, *^4^J* = 1.3 Hz, Ar-H), 7.86 (m, 1H, Ar-H), 9.22 (dd, 1H, *^3^J* = 8.3 Hz, *^4^J* = 1.0 Hz, Ar-H), 11.91 (s, 1H, NH), 14.49 (s, 1H, NH). ^13^C-NMR: δ 27.2 (3C, COC(CH_3_)_3_), 40.6 (COC(CH_3_)_3_), 117.4, 122.4, 123.6, 123.7, 124.4, 127.2, 129.4, 136.8, 138.3, 145.3, 147.4 (aromatic-C), 161.2 (CN_3_), 180.2 (C=O). Anal. Calcd. for C_17_H_19_ClN_4_O (330.81): C, 61.72;H, 5.79; N, 16.96. Found: C, 61.49; H, 5.72; N, 16.99%.

*N-Pivaloyl-N′-(3-chlorophenyl)-N′′-pyridylguanidine* (**3**). Compound **3** was prepared and purified in the same way as **1**, using *N*-pivaloyl-*N′*-pyridylthiourea (2.37 g, 10 mmol), 3-chloroaniline (1.1 mL, 10 mmol), triethylamine (2.8 mL, 20 mmol) and mercury(II) chloride (2.72 g, 10 mmol). Yield: 2.45 g, 74%. Colourless crystals. M. p. 67–68 °C. FT-IR (cm^−1^): 3427, 3254, 3142, 3052, 2965, 1627, 1555, 1460, 1373, 1202, 834, 757. ^1^H-NMR: δ 1.12 (s, 9H, COC(CH_3_)_3_), 6.37–6.42 (m, 1H, Ar-H), 6.78–6.86 (m, 1H, Ar-H), 6.89–6.93 (m, 1H, Ar-H), 7.00–7.10 (m, 2H, Ar-H), 7.32–7.36 (m, 1H, Ar-H), 7.83–7.85 (m, 1H, Ar-H), 8.50 (s, 1H, Ar-H), 11.32 (s, 1H, NH), 14.46 (s, 1H, NH). ^13^C-NMR: δ 27.3 (3C, COC(CH_3_)_3_), 40.7 (COC(CH_3_)_3_), 117.5, 119.2, 121.6, 122.7, 123.5, 130.0, 134.9, 138.5, 140.7, 145.3, 147.2 (aromatic-C), 161.3 (CN_3_), 180.9 (C=O). Anal. Calcd. for C_17_H_19_ClN_4_O (330.81): C, 61.72; H, 5.79; N, 16.96. Found: C, 61.58; H, 5.83; N, 16.87%.

*N-Pivaloyl-N′-(4-chlorophenyl)-N′′-pyridylguanidine* (**4**). Compound **4** was prepared and purified in the same way as **1**, using *N*-pivaloyl-*N′*-pyridylthiourea (2.37 g, 10 mmol), 4-chloroaniline (1.28 g, 10 mmol), triethylamine (2.8 mL, 20 mmol) and mercury(II) chloride (2.72 g, 10 mmol). Yield: 2.38 g, 72%. Colourless crystals. M. p. 82–83 °C. FT-IR (cm^−1^): 3411, 3259, 3137, 3074, 2962, 1617, 1552, 1445, 1381, 1067, 872. ^1^H-NMR: δ 1.14 (s, 9H, COC(CH_3_)_3_), 6.39–6.43 (m, 1H, Ar-H), 6.97–7.02 (m, 1H, Ar-H), 7.07–7.11 (m, 1H, Ar-H), 7.13 (d, 2H, *^3^J* = 8.9 Hz, Ar-H), 7.69 (d,, 2H, *^3^J* = 8.9 Hz, Ar-H),7.84–7.87 (m, 1H, Ar-H), 11.26 (s, 1H, NH), 14.48 (s, 1H, NH). ^13^C-NMR: δ 27.2 (3C, COC(CH_3_)_3_), 40.6 (COC(CH_3_)_3_), 117.2, 122.4, 122.6 (2C), 123.8, 129.0 (2C), 137.9, 138.3, 145.2, 147.2 (aromatic-C), 161.3 (CN_3_), 180.7 (C=O). Anal. Calcd. For C_17_H_19_ClN_4_O (330.81): C, 61.72; H, 5.79; N, 16.94. Found: C, 61.63; H, 5.65; N, 17.01%.

*N-Pivaloyl-N′-(2-methoxyphenyl)-N′′-pyridylguanidine* (**5**). Compound **5** was prepared and purified in the same way as **1**, using *N*-pivaloyl-*N′*-pyridylthiourea (2.37 g, 10 mmol), *o*-anisidine (1.14 mL, 10 mmol), triethylamine (2.8 mL, 20 mmol) and mercury(II) chloride (2.72 g, 10 mmol). Yield: 2.48 g, 76%. Colourless crystals. M. p. 80–81 °C. FT-IR (cm^−1^): 3424, 3247, 3162, 3022, 2964, 1648, 1543, 1467, 1380, 1104, 848, 729. ^1^H-NMR: δ 1.15 (s, 9H, COC(CH_3_)_3_), 3.39 (s, 3H, OCH_3_), 6.38–6.43 (m, 1H, Ar-H), 6.56 (d, 1H, *^3^J* = 8.1 Hz, Ar-H), 6.91–6.96 (m, 1H, Ar-H), 7.09–7.13 (m, 3H, Ar-H), 7.87–7.90 (m, 1H, Ar-H), 9.47 (dd, 1H, *^3^J* = 8.1 Hz, *^4^J* = 1.6 Hz, Ar-H), 11.93 (s, 1H, NH), 14.54 (s, 1H, NH). ^13^C-NMR: δ 27.3 (3C, COC(CH_3_)_3_), 40.6 (COC(CH_3_)_3_), 55.5 (OCH_3_), 110.3, 116.8, 121.1, 121.9, 122.5, 123.0, 129.5, 138.2, 145.2, 147.6, 149.6 (aromatic-C), 161.8 (CN_3_), 179.9 (C=O). Anal. Calcd. for C_18_H_22_N_4_O_2_ (326.39): C, 66.24; H, 6.79; N, 17.17. Found: C, 66.01; H, 6.82; N, 16.96%.

*N-Pivaloyl-N′-(4-tolyl)-N′′-pyridylguanidine* (**6**). Compound **6** was prepared and purified in the same way as **1**, using *N*-pivaloyl-*N′*-pyridylthiourea (2.37 g, 10 mmol), *p*-toluidine (1.07 g, 10 mmol), triethylamine (2.8 mL, 20 mmol) and mercury(II) chloride (2.72 g, 10 mmol). Yield: 2.14 g, 69%. Colourless crystals. M. p. 67–68 °C. FT-IR (cm^−1^): 3419, 3252, 3148, 3037, 2951, 1627, 1534, 1462, 1379, 1237, 928, 769. ^1^H-NMR: δ 1.15 (s, 9H, COC(CH_3_)_3_), 2.12 (s, 3H, Ar-CH_3_), 6.39–6.43 (m, 1H, Ar-H), 7.01–7.11 (m, 4H, Ar-H), 7.88–7.92 (m, 3H, Ar-H), 11.36 (s, 1H, NH), 14.53 (s, 1H, NH). ^13^C-NMR: δ 20.9 (Ar-CH_3_), 27.2 (3C, COC(CH_3_)_3_), 40.6 (COC(CH_3_)_3_), 116.8, 121.5 (2C), 122.3, 129.6 (2C), 132.8, 137.0, 138.2, 145.2, 147.6 (aromatic-C), 161.8 (CN_3_), 180.6 (C=O). Anal. Calcd. for C_18_H_22_N_4_O (310.39): C, 69.65; H, 7.14; N, 18.05. Found: C, 69.34; H, 7.11; N, 18.21%.

*N-Pivaloyl-N′-(2-fluorophenyl)-N′′-pyridylguanidine* (**7**). Compound **7** was prepared and purified in the same way as **1**, using *N*-pivaloyl-*N′*-pyridylthiourea (2.37 g, 10 mmol), 2-fluoroaniline (1.0 mL, 10 mmol), triethylamine (2.8 mL, 20 mmol) and mercury(II) chloride (2.72 g, 10 mmol). Yield: 2.20 g, 70%. Colourless crystals. M. p. 80–81 °C. FT-IR (cm^−1^): 3428, 3243, 3140, 3062, 2957, 1617, 1564, 1454, 1363, 1055, 728. ^1^H-NMR: δ 1.12 (s, 9H, COC(CH_3_)_3_), 6.39–6.42 (m, 1H, Ar-H), 6.65–6.70 (m, 1H, Ar-H), 6.83–6.88 (m, 1H, Ar-H), 7.01–7.05 (m, 2H, Ar-H), 7.07–7.12 (m, 1H, Ar-H), 7.85–7.87 (m, 1H, Ar-H), 9.13–9.17 (m, 1H, Ar-H), 11.79 (s, 1H, NH), 14.47 (s, 1H, NH). ^13^C-NMR: δ 27.2 (3C, COC(CH_3_)_3_), 40.6 (COC(CH_3_)_3_), 114.7, 117.3, 122.4, 123.2, 123.3, 123.4, 124.3, 124.4, 138.3, 145.3, 147.4 (aromatic-C), 161.3 (CN_3_), 180.5 (C=O). Anal. Calcd. for C_17_H_19_N_4_OF (314.36): C, 64.95; H, 6.09; N, 17.82. Found: C, 64.77; H, 6.14; N, 17.73%.

*N-Pivaloyl-N′-(n-propyl)-N′′-pyridylguanidine* (**8**). Compound **8** was prepared and purified in the same way as **1**, using *N*-pivaloyl-*N′*-pyridylthiourea (2.37 g, 10 mmol), *n*-propylamine (0.8 mL, 10 mmol), triethylamine (2.8 mL, 20 mmol) and mercury(II) chloride (2.72 g, 10 mmol). Yield: 2.07 g, 79%. Colourless crystals. M. p. 61–62 °C. FT-IR (cm^−1^): 3437, 3268, 3074, 2986, 1619, 1534, 1435, 1395, 1238, 958, 857. ^1^H-NMR: δ 0.86 (t, 3H, *^3^J* = 7.4, NCH_2_CH_2_CH_3_), 1.22 (s, 9H, COC(CH_3_)_3_), 1.41–1.55 (m 2H, NCH_2_CH_2_CH_3_), 3.37–3.47 (m, 2H, NCH_2_CH_2_CH_3_), 6.48–6.51 (m, 1H, Ar-H), 7.14–7.16 (m, 1H, Ar-H), 7.20–7.25 (m, 1H, Ar-H), 7.96–7.98 (m, 1H, Ar-H), 9.02 (s, 1H, NH), 14.59 (s, 1H, NH). ^13^C-NMR: δ 11.7 (NCH_2_CH_2_CH_3_), 22.9 (NCH_2_CH_2_CH_3_), 27.3 (3C, COC(CH_3_)_3_), 40.5 (COC(CH_3_)_3_), 42.7 (NCH_2_CH_2_CH_3_), 115.8, 121.7, 138.0, 145.1, 145.9 (aromatic-C), 162.7 (CN_3_), 180.3 (C=O). Anal. Calcd. for C_14_H_22_N_4_O (262.35): C, 64.09; H, 8.45; N, 21.36. Found: C, 63.92; H, 8.40; N, 21.27%.

*N-Pivaloyl-N′-(iso-propyl)-N′′-pyridylguanidine* (**9**). Compound **9** was prepared and purified in the same way as **1**, using *N*-pivaloyl-*N′*-pyridylthiourea (2.37 g, 10 mmol), isopropylamine (0.9 mL, 10 mmol), triethylamine (2.8 mL, 20 mmol) and mercury(II) chloride (2.72 g, 10 mmol). Yield: 2.10 g, 80%. Colourless crystals. M. p. 65–66 °C. FT-IR (cm^−1^): 3428, 3239, 3143, 3057, 2964, 1627, 1554, 1483, 1353, 1232, 927, 785. ^1^H-NMR: δ 1.15 (d, 6H, *^3^J* = 6.6 Hz, NCH(CH_3_)_2_), 1.18 (s, 9H, COC(CH_3_)_3_), 4.35–4.53 (m, 1H, NCH(CH_3_)_2_), 6.18–6.27 (m, 1H, Ar-H), 6.42–6.45 (m, 1H, Ar-H), 7.12–7.16 (m, 1H, Ar-H), 7.92–7.95 (m, 1H, Ar-H), 8.99 (s, 1H, NH), 14.57 (s, 1H, NH). ^13^C-NMR: δ 22.8 (2C, NCH(CH_3_)_2_), 27.3 (3C, COC(CH_3_)_3_), 40.5 (COC(CH_3_)_3_), 42.4 (NCH(CH_3_)_2_), 115.8, 121.7, 138.0, 145.1, 150.3 (aromatic-C), 162.7 (CN_3_), 180.4 (C=O). Anal. Calcd. for C_14_H_22_N_4_O (262.35): C, 64.09; H, 8.45; N, 21.36. Found: C, 63.87; H, 8.47; N, 21.25%.

*N-Pivaloyl-N′-(iso-butyl)-N′′-pyridylguanidine* (**10**). Compound **10** was prepared and purified in the same way as **1**, using *N*-pivaloyl-*N′*-pyridylthiourea (2.37 g, 10 mmol), *iso*-butylamine (1.1 mL, 10 mmol), triethylamine (2.8 mL, 20 mmol) and mercury(II) chloride (2.72 g, 10 mmol). Yield: 2.16 g, 78%). Colourless crystals. M. p. 56–57 °C. FT-IR (cm^−1^): 3429, 3258, 3148, 3071, 2964, 1628, 1558, 1461, 1383, 1126, 992, 843, 761. ^1^H-NMR: δ 0.88 (t, 3H, *^3^J* = 7.5 Hz, NCH(CH_3_)CH_2_CH_3_), 1.15 (s, 9H, COC(CH_3_)_3_), 1.35–1.58 (m, 5H, NCH(CH_3_)CH_2_CH_3_), 4.37–4.40 (m, 1H, NCH(CH_3_)CH_2_CH_3_), 6.41–6.45 (m, 1H, Ar-H), 7.08–7.10 (m, 1H, Ar-H), 7.14–7.19 (m, 1H, Ar-H), 7.90–7.92 (m, 1H, Ar-H), 8.98 (s, 1H, NH), 14.56 (s, 1H, NH). ^13^C-NMR: δ 10.5 (NCH(CH_3_)CH_2_CH_3_), 27.3 (3C, COC(CH_3_)_3_), 28.6 (NCH(CH_3_)CH_2_CH_3_), 29.7 (NCH(CH_3_)CH_2_CH_3_), 40.6 (COC(CH_3_)_3_), 47.7 (NCH(CH_3_)CH_2_CH_3_), 115.8, 121.7, 138.0, 145.1, 145.9 (aromatic-C), 162.7 (CN_3_), 180.4 (C=O). Anal. Calcd. for C_15_H_24_N_4_O (276.38): C, 65.19; H, 8.75; N, 20.27. Found: C, 64.91; H, 8.80; N, 20.19%. 

*N-Pivaloyl-N′,N′′-bipyridylguanidine* (**11**). Compound **11** was prepared and purified in the same way as **1**, using *N*-pivaloyl-*N′*-pyridylthiourea (2.37 g, 10 mmol), 2-aminopyridine (0.94 g, 10 mmol), triethylamine (2.8 mL, 20 mmol) and mercury(II) chloride (2.72 g, 10 mmol). Yield: 2.26 g, 76%. Colourless crystals. M. p. 90–91 °C. FT-IR (cm^−1^): 3423, 3263, 3069, 2943, 1618, 1539, 1448, 1371, 1172, 937, 861. ^1^H-NMR: δ 1.10 (s, 9H, COC(CH_3_)_3_), 6.40–6.44 (m, 1H, Ar-H), 6.52–6.56 (m, 1H, Ar-H), 7.02–7.12 (m, 2H, Ar-H), 7.31–7.37 (m, 1H, Ar-H), 7.84–7.86 (m, 1H, Ar-H), 8.26–8.28 (m, 1H, Ar-H), 8.96 (d, 1H, *^3^J* = 8.4 Hz, Ar-H), 12.08 (s, 1H, NH), 14.36 (s, 1H, NH). ^13^C-NMR: δ 27.1 (3C, COC(CH_3_)_3_), 40.7 (COC(CH_3_)_3_), 115.3, 117.4, 118.8, 122.3, 137.4, 138.3, 145.3, 147.0, 148.7, 152.9 (aromatic-C), 161.3 (CN_3_), 180.0 (C=O). Anal. Calcd. for C_16_H_19_N_5_O (297.35): C, 64.63; H, 6.44; N, 23.55. Found: C, 64.31; H, 6.37; N, 23.42%.

*N-Pivaloyl-N′,N′-dipropyl-N′′-pyridylguanidine* (**12**). Compound **12** was prepared and purified in the same way as **1**, using *N*-pivaloyl-*N′*-pyridylthiourea (2.37 g, 10 mmol), dipropylamine (1.38 mL, 10 mmol), triethylamine (2.8 mL, 20 mmol) and mercury(II) chloride (2.72 g, 10 mmol). Yield: 2.28 g, 75%. Colourless crystals. M. p. 80–81 °C. FT-IR (cm^−1^): 3439, 3142, 3057, 2972, 1658, 1549, 1481, 1361, 1237, 976, 775. ^1^H-NMR: δ 0.86 (t, 6H, *^3^J* = 7.3 Hz, N(CH_2_CH_2_CH_3_)_2_), 1.15 (s, 9H, COC(CH_3_)_3_), 1.61 (sext, 4H, *^3^J* = 7.3, N(CH_2_CH_2_CH_3_)_2_), 3.36–3.38 (m, 4H, N(CH_2_CH_2_CH_3_)_2_), 6.40–6.44 (m, 1H, Ar-H), 6.96–6.98 (m, 1H, Ar-H), 7.07–7.12 (m, 1H, Ar-H), 8.04–8.05 (m, 1H, Ar-H), 12.27 (s, 1H, NH). ^13^C-NMR: δ 11.6 (2C, N(CH_2_CH_2_CH_3_)_2_), 21.6 (2C, N(CH_2_CH_2_CH_3_)_2_), 27.4 (3C, COC(CH_3_)_3_), 40.3 (COC(CH_3_)_3_), 50.6 (2C, N(CH_2_CH_2_CH_3_)_2_), 116.4, 121.2, 137.8, 146.2, 150.4 (aromatic-C), 161.8 (CN_3_), 176.7 (C=O). Anal. Calcd. for C_17_H_28_N_4_O (304.43): C, 67.07; H, 9.27; N, 18.40. Found: C, 66.89; H, 9.18; N, 18.37%.

### 3.3. Biological Screening Protocols

#### 3.3.1. Potato Disc Anti-Tumor Assay

Guanidine derivatives were evaluated for antitumor properties using the potato disc bioassay described by Turker and Camper [42]. Sterilized potatoes (*Solanum tuberosum*) were cut into 5 mm × 8 mm in size from the center of potato tissue by sterilize cork borer. Each potato disc was loaded with 50 µL of appropriate inoculums. Each inoculum was prepared by taking 150 µL of test sample (5000 µg/mL in DMSO), 750 µL autoclaved distilled water and 600 µL *Agrobacterium tumefaciens* (AT10) in PBS. After inoculation, Petri dishes were sealed by Parafilm^®^ and incubated at 27–30 °C for 3 weeks. Tumors were observed on potato discs under stereo microscope after 30 min of staining it with Lugol’s solution (10% KI and 5% I_2_). Number of tumors per disc was counted and percentage inhibition was calculated for triplicate experiment. 

Three controls were used in the assay:

a. Positive Control; prepared by taking 150 µL of DMSO in autoclaved Eppendorfs and then adding 1,350 µL of autoclaved distilled water;
b. Negative control; prepared by taking 150 µL of DMSO in autoclaved Eppendorfs, then adding 750 µL of autoclaved distilled water and 600 µL of bacterial culture;
c. Blank potato discs used as control.

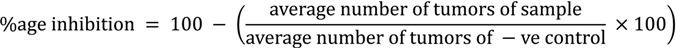



#### 3.3.2. Anti-Oxidant Study

The anti-oxidant behavior of synthesized compounds was investigated by reported method of Siraj *et al*. [43] with a few modifications. For the modified procedure, stock solutions of DPPH and samples were prepared in 80% methanol. Test samples were prepared by mixing calculated volume of samples stock solutions and DPPH stock solution. Final concentration of samples were kept in range of 14–1,000 μg while fixed amount of DPPH was added to all samples in such a way that mixture had absorbance around 0.99 at 517 nm at the time of mixing. Samples were prepared in triplicate for each concentration used and at least seven different concentrations were used for each sample. The sample tubes were covered with aluminum foils and left in incubator at 37 °C. After 1, 24, 48 and 72 h, the absorbance at 517 nm was recorded by UV-V is spectrophotometer. DPPH solution was used as a control. The scavenging activity was estimated which is based on the percentage of DPPH radical scavenged, using the following equation:

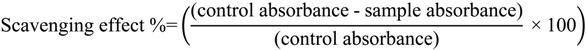



#### 3.3.3. Antifungal Activity

The synthesized guanidines were also investigated for their antifungal activity against five fungal strains *i.e.*, *Aspergillus flavus*, *Aspergillus niger*, *Fusarium solanai*, *Mucor species* and *Aspergillus fumagatus*. Susceptibility test was performed by using the agar tube dilution method [53] with some modifications and using terbinafine as reference drug. Screw capped test tubes containing Sabouraud Dextrose Agar (SDA) medium (4 mL) were autoclaved at 121 °C for 15 min. Tubes were allowed to cool at 50 °C and non solidified SDA was loaded with 66.6 µL of test compound from the stock solution (12 mg/mL in DMSO) to make 200 µg/mL final concentration. Tubes were then allowed to solidify in slanting position at room temperature. Each tube was inoculated with 4 mm diameter piece of inoculum from seven days old fungal culture. The media supplemented with DMSO and terbinafine (200 µg/mL) were used as negative and positive control, respectively. The tubes were incubated at 28 °C for 7 d and growth in the media was determined by measuring linear growth (mm). Growth inhibition was calculated with reference to growth in vehicle control as shown in equation.

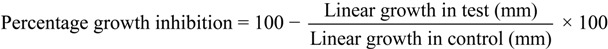



More than 70% inhibition was considered as significant, 60%–70% as good, 50%–60% as moderate and below 50% as insignificant activity.

#### 3.3.4. Antibacterial Activity

The synthesized compounds were tested against six bacterial strains; two Gram-Positive [*Micrococcus luteus* (ATCC10240) and *Staphylococcus aureus* (ATCC6538)] and four Gram-negative [*Klebsiella pneumoniae* (MTCC618), *Enterobactor aerogenes* (ATCC13048), *Escherichia coli* (ATCC15224) and *Bordetella bronchiseptica* (ATCC4617)]. The agar well-diffusion method [44] was used for the determination of inhibition zones and minimum inhibitory concentration (MIC). Broth culture (0.75 mL) containing ca. 10^6^ colony forming units (CFU) per mL of the test strain was added to 75 mL of nutrient agar medium at 45 °C, mixed well, and then poured into a 14 cm diameter sterile Petri plate. The media was allowed to solidify and 8 mm wells were dug with a sterile metallic borer. Then a DMSO solution of test sample (100 µL) at 1 mg/mL was added to the respective wells. DMSO served as negative control, and the standard antibacterial drug cefixime (1 mg/mL) and roxyithromycin (1 mg/mL) were used as positive control. Triplicate plates of each bacterial strain were prepared which were incubated aerobically at 37 °C for 24 h. The activity was determined by measuring the diameter of zone showing complete inhibition (mm). Thereby zones were precisely measured with the aid of a Vernier caliper (precision ± 0.1 mm). The growth inhibition was calculated with reference to the positive control.

## 4. Conclusions

A series of *N*-pivaloyl-*N′*-(alkyl/aryl)-*N″*-pyridylguanidine of general formula (C_4_H_9_CONHC(NR^1^R^2^)NPy), (where R^1^ = aryl/alkyl group, R^2^ = H **1**–**11** and *n*-propyl (compound **12**) and Py = 2-pyridyl group) were synthesized and characterized. IR, NMR spectroscopy and XRD studies revealed that synthesized guanidines are stabilized by intramolecular hydrogen bonds. All the compounds showed excellent inhibition against *Agrobacterium tumefaciens* (AT10) induced tumor. It is observed that the aryl substituted pyridylguanidines are more potent anti-tumor agents compared with the alkyl substituted compounds. The antioxidant studies showed that the presence of electron donor substituents such as alkyl group at N′ position enhances the DPPH scavenging ability. The synthesized compounds revealed insignificant antibacterial activities, but a few compounds exhibited good antifungal properties.
